# The Influence of Attribution Style and Goal Accessibility on Health Beliefs and Exercise Willingness: Experimental Evidence from University Students

**DOI:** 10.3390/bs15060763

**Published:** 2025-06-02

**Authors:** Shuai Zhang, Chenglong Miao

**Affiliations:** 1 Department of Leisure Sports, Kangwon National University, Samcheok 25913, Republic of Korea; zhangsh89889@gmail.com; 2 Institute of Sport Psychology Techniques and Training, Kangwon National University, Samcheok 25913, Republic of Korea

**Keywords:** perceived goal attainability, causal attribution, motivation for exercise, health behavior theory, attributional reappraisal, psychological intervention

## Abstract

Although the benefits of regular physical activity are widely recognized, many university students fail to sustain consistent exercise behaviors. This phenomenon may be attributed to cognitive and motivational barriers, particularly perceptions of goal attainability and attribution styles, which are believed to significantly influence students’ health beliefs and intentions to engage in physical activity. This research aimed to examine the independent and combined effects of goal attainability and attribution style on Chinese university students’ health beliefs and willingness to exercise. The study also investigated how shifts in attribution style may influence these outcomes under different levels of goal attainability. Two between-subjects experiments were conducted. In Experiment 1 (N = 146), a 2 (goal attainability: high vs. low) × 2 (attribution style: internal vs. external) design was used. Participants were exposed to tailored exercise advertisements and completed standardized questionnaires measuring health beliefs and exercise intentions. Experiment 2 (N = 130) adopted a 2 (goal attainability: high vs. low) × 2 (attributional shift: external-to-internal vs. internal-to-external) design, utilizing visual priming and short video interventions to manipulate attributional orientation. In Experiment 1, both high goal attainability and internal attribution independently enhanced participants’ health beliefs and exercise willingness. A significant interaction effect was observed only for exercise willingness, with the highest intentions found in the high attainability × internal attribution group. In Experiment 2, shifting attribution from external to internal significantly increased both health beliefs and exercise willingness, while shifting from internal to external resulted in substantial decreases. An interaction effect was again found only for exercise willingness, suggesting that the effectiveness of attributional shift depended on goal attainability. By integrating the Health Belief Model with Attribution Theory, this study offers a deeper understanding of how cognitive and motivational factors influence exercise behavior, and provides a theoretical foundation for developing adaptive interventions.

## 1. Introduction

With the shift in modern societal lifestyles, the relationship between physical exercise and health has gained increasing attention. Numerous studies have shown that regular physical exercise not only improves physical health and delays aging, but also significantly enhances psychological well-being and overall quality of life (Haluza et al., 2025). Despite widespread recognition of the various benefits of exercise, many individuals still fail to develop regular exercise habits, with participation rates falling far below expectations. This phenomenon is particularly prominent among young people, especially college students, who often face multiple challenges, including academic and life pressures, which limit their exercise intentions and, consequently, hinder the actual implementation of exercise behaviors.

In this context, exploring the psychological factors that influence health beliefs and exercise intentions among college students has become a critical issue in health promotion research. Goal accessibility and attribution style are key psychological factors that affect individuals’ health beliefs and exercise intentions. The Health Belief Model (HBM) posits that an individual’s intention to engage in health behaviors is influenced by multiple factors, including perceived health threats, the benefits of the behavior, and potential barriers (Rosenstock, 1974; Taira et al., 2025). Attribution theory, on the other hand, emphasizes that the attributions individuals make about the outcomes of their behavior determine their motivation and the persistence of their actions (Weiner, 1985; Brun et al., 2021). Specifically, whether individuals perceive the results of exercise as dependent on personal effort (internal attribution) or external factors (external attribution) directly influences their exercise intentions and motivation to continue participation.

However, although existing studies have explored the impact of goal accessibility and attribution style on health beliefs and exercise intentions, these studies typically examine each factor in isolation, with limited focus on the interaction between these factors and their specific mechanisms of influence on health beliefs and exercise intentions. In particular, how attribution style influences individuals’ health beliefs and exercise intentions under different levels of goal accessibility remains underexplored in systematic empirical research. To address these gaps, the present study aims to systematically examine how goal accessibility and attribution style—both independently and interactively—influence university students’ health beliefs and exercise intentions. Specifically, it explores whether higher perceived goal attainability enhances motivation and belief in health-related behavior, whether internal attribution fosters stronger behavioral intentions than external attribution, and whether the combination of these two psychological factors jointly shapes exercise-related decision-making. To this end, a controlled experimental design was employed to test these relationships and provide theoretical insights and practical implications for exercise intervention strategies.

## 2. Literature Review and Research Hypotheses

### 2.1. The Impact of Goal Accessibility on Health Beliefs and Exercise Intentions

Goal accessibility refers to an individual’s perception and cognition regarding the attainability of a particular goal (Strauman & Eddington, 2017). Research has shown that an individual’s perception of goal accessibility plays a significant role in shaping their health beliefs and exercise intentions. When individuals perceive exercise goals as achievable, they are more likely to form positive expectations about the outcomes of exercise, thereby increasing their confidence in the health benefits that exercise can provide (Sokolova & Perez, 2021; Durau et al., 2022). For instance, when individuals believe that exercise can yield health improvements in a short period, they are more inclined to believe in its significant effects on physical health, thereby strengthening their health beliefs. In contrast, if individuals perceive exercise goals as distant or difficult to attain, with health benefits taking a long time to materialize, such perceptions may lead to doubts about the effectiveness of exercise, thus weakening their health beliefs (Katz & Bosworth, 2016). Therefore, goal accessibility not only directly influences an individual’s trust and expectations regarding the effects of exercise but also indirectly determines the strength of their health beliefs.

Furthermore, the impact of goal accessibility on exercise intentions is similarly significant. When individuals perceive exercise goals as attainable, they typically exhibit stronger motivation to exercise and demonstrate higher levels of engagement in actual behavior (Schunk & DiBenedetto, 2020). This perception of goal accessibility stimulates intrinsic motivation, encouraging individuals to actively participate in exercise and persist in their efforts when faced with challenges. In contrast, if individuals perceive exercise goals as too distant or difficult to achieve, they may feel that the goals are unattainable, leading to a decline in motivation and even the emergence of exercise avoidance behaviors (Plys et al., 2020). Thus, goal accessibility not only influences the intensity of exercise motivation by shaping individuals’ expectations regarding exercise goals, but also directly affects their willingness to participate in exercise and the persistence of their behavior.

### 2.2. The Impact of Attribution Style on Health Beliefs and Exercise Intentions

Attribution style refers to the tendency of individuals to explain the outcomes of behaviors through internal or external attributions (Perez-Rodriguez et al., 2015), playing a significant psychological role in shaping health beliefs and exercise-related decision-making. The impact of attribution style on health beliefs is primarily reflected in the construction of an individual’s sense of control and self-efficacy (Ruan et al., 2023). When individuals attribute exercise outcomes to internal factors, such as their own effort, ability, or perseverance, they tend to develop a stronger sense of behavioral control, thereby enhancing their confidence in the effects of exercise (Zhao et al., 2024). This internal attribution pattern strengthens the perception of controllability over exercise outcomes, making individuals more likely to believe in the tangible and positive health benefits of physical activity. For instance, individuals who hold the belief that “as long as I persist in exercising, my health will improve” are more likely to develop stable and robust health beliefs. In contrast, when individuals adopt an external attribution style, attributing exercise outcomes to uncontrollable factors such as the environment, weather, or genetic predispositions, their sense of control over the results significantly diminishes, thereby weakening their recognition and trust in the health benefits of exercise and leading to instability in their health beliefs.

The impact of attribution style on exercise willingness is equally crucial. According to motivation attribution theory, internal attributions can stimulate higher levels of intrinsic motivation, as individuals believe that their actions can drive outcomes, thereby enhancing their willingness to sustain behavior (Xu et al., 2020; Riley, 2016). Specifically, when individuals attribute exercise results primarily to their own effort or ability, they are more likely to invest time and energy in maintaining or improving their exercise levels. In contrast, external attributions often lead to a sense of helplessness, as individuals may feel that their efforts are ineffective, thereby reducing motivation for exercise and decreasing both the frequency and persistence of the behavior (Frazier et al., 2021). Therefore, attribution style, by influencing an individual’s perception of control over exercise outcomes, indirectly determines the strength of their exercise willingness. Overall, attribution style is not only an important variable influencing an individual’s belief system but also a core psychological element in regulating their motivational mechanisms.

### 2.3. The Interaction Between Goal Accessibility and Attribution Style and Its Impact on Health Beliefs and Exercise Willingness

Although goal accessibility and attribution style are independent psychological variables, their interaction plays a crucial role in shaping an individual’s health beliefs and exercise willingness. The combination of an individual’s perception of goal accessibility and their attribution style determines their belief in exercise outcomes and their continued engagement in physical activity (Granero-Gallegos et al., 2017). When individuals perceive exercise goals as attainable and attribute outcomes to their own efforts, this combination typically strengthens both their health beliefs and exercise willingness (Schunk & DiBenedetto, 2021). The synergy of high goal accessibility and internal attribution leads individuals not only to believe that exercise goals are achievable, but also to think that they can control exercise results through their efforts, thus enhancing trust in exercise and fostering positive expectations regarding health outcomes (Heng et al., 2025; Khuyen et al., 2023). This perception further boosts individuals’ confidence in the benefits of exercise, making them more resolute in their belief that physical activity can effectively improve health.

In contrast, when individuals perceive exercise goals as difficult to achieve and attribute outcomes to external factors (such as weather, environment, or other uncontrollable elements), they may develop lower health beliefs and exercise willingness (Browall et al., 2018; Bijoux Leist & Leist, 2022; Boddu et al., 2021). External attributions typically lead to a diminished sense of control over exercise outcomes, which weakens their expectations of exercise effects and may even result in avoidance behaviors toward exercise (Esmaeilzadeh et al., 2022). The combination of low goal accessibility and external attribution often undermines individuals’ confidence in exercise, leading them to believe that exercise results cannot be effectively controlled through personal effort. As a result, both their health beliefs and exercise willingness are significantly inhibited.

This interaction mechanism suggests that different combinations of goal accessibility and attribution style can have a profound impact on individuals’ health beliefs and exercise willingness. The combination of high goal accessibility and internal attribution not only enhances an individual’s sense of self-efficacy but also significantly boosts their trust in health behaviors and their intention to engage in them. Conversely, the combination of low goal accessibility and external attribution tends to diminish an individual’s sense of control and weaken their behavioral motivation (Alrobai et al., 2019). Therefore, understanding the dynamic relationship between goal accessibility and attribution style is of significant theoretical importance and practical value for interventions targeting health beliefs and motivating exercise willingness.

### 2.4. The Impact of Attribution Style Shift on Health Beliefs and Exercise Willingness

Attribution style shift refers to the change in an individual’s attribution of exercise outcomes from external factors to internal factors, or vice versa (Zuelke & Afful, 2023). Research indicates that attribution style shift has a significant impact on health beliefs and exercise willingness, particularly in enhancing the sense of control and self-efficacy. When individuals attribute exercise outcomes to their own efforts (i.e., shifting from external to internal attribution), they typically experience an increased sense of control over the results, thereby strengthening their belief in the benefits of exercise (Hulleman et al., 2017). When individuals perceive exercise outcomes as determined by personal effort and ability, they become more convinced that exercise can effectively improve their health, thus reinforcing their health beliefs. This not only enhances self-efficacy but also increases recognition of exercise, fostering an intention to engage in physical activity.

Conversely, when individuals attribute exercise outcomes to external factors (such as weather, environment, or other uncontrollable elements), they may experience a lack of control, weakening their belief in exercise outcomes and their motivation to participate (Tao et al., 2023). External attributions create uncertainty regarding exercise effects, as individuals perceive their outcomes to be highly influenced by external conditions, which diminishes their exercise belief. This suggests that attribution style shift plays a crucial role in the formation of health beliefs, with an individual’s exercise motivation and behavioral persistence often depending on their attribution style.

Additionally, the impact of attribution style shift on exercise willingness is also significant. When external attributions are shifted to internal attributions, individuals’ willingness to engage in exercise typically increases significantly (Huang et al., 2025). This shift enhances self-efficacy, improves expectations and confidence in exercise outcomes, and stimulates stronger intrinsic motivation, driving more active participation in exercise. Conversely, when internal attributions are shifted to external attributions, individuals may feel a lack of control, leading to a decline in exercise willingness. External attributions reduce intrinsic motivation, as individuals believe that external factors determine exercise outcomes, thereby diminishing their drive to maintain exercise.

Therefore, attribution style shift not only influences individuals’ perceptions and beliefs about exercise outcomes but also profoundly affects their exercise willingness and the sustainability of their behavior. By shifting attribution styles, enhancing self-efficacy, and fostering trust in the benefits of exercise, individuals’ willingness to exercise and their health beliefs can be significantly strengthened, promoting more active participation and sustained engagement in physical activity. Attribution style shift provides an important theoretical foundation for exercise interventions, particularly in enhancing exercise participation and promoting health behaviors.

### 2.5. Research Hypotheses

Based on research related to the HBM and Attribution Theory, an individual’s health beliefs and exercise willingness are influenced by multiple factors, including goal accessibility and attribution style. The HBM posits that individuals’ intentions to engage in health behaviors are influenced by their perceived health threats, the benefits of the behavior, and potential barriers (Sheeran et al., 2020). Goal accessibility, as a crucial factor in an individual’s perception of health goal attainability, may, to some extent, influence their health beliefs and exercise willingness (Yeager & Dweck, 2020). Attribution Theory emphasizes that an individual’s attribution of exercise outcomes to internal factors (such as personal effort) or external factors (such as environment or genetics) directly impacts their health beliefs and exercise willingness (Miller et al., 2017). A shift in attribution style, specifically from external to internal attributions, typically leads to a significant increase in individuals’ health beliefs and exercise willingness (Hovenkamp-Hermelink et al., 2019; Luszczynska & Schwarzer, 2005; Pourhoseinzadeh et al., 2017).

Although previous research has explored the independent effects of goal accessibility and attribution style on health beliefs and exercise willingness, few studies have delved into the interaction between these two factors, and the further impact of attribution style shift on health beliefs and exercise willingness under varying goal accessibility conditions. Therefore, this study designed Experiment 1 and Experiment 2, proposing the following hypotheses, aiming to explore how goal accessibility and attribution style jointly influence individuals’ health beliefs and exercise willingness, and to verify their interaction:

Hypotheses for Experiment 1:

**Hypothesis 1** **(H1):** *The higher the perceived attainability of the goal, the stronger an individual’s health beliefs and exercise intentions*.

**Hypothesis 2** **(H2):** *Individuals who adopt an internal attribution style exhibit stronger health beliefs and greater exercise intentions compared to those who adopt an external attribution style*.

**Hypothesis 3** **(H3):** *There is an interaction between goal attainability and attribution style in shaping health beliefs and exercise intentions. If H3 is supported, further testing of H4 will proceed; if H3 is not supported, H4 will be discarded*.

**Hypothesis 4** **(H4a):** *If H3 is supported, individuals with high goal attainability and an internal attribution style will exhibit the strongest health beliefs, while individuals with low goal attainability and an external attribution style will exhibit the weakest health beliefs*.

**Hypothesis 4b** **(H4b):** *If H3 is supported, individuals with high goal attainability and an internal attribution style will demonstrate the strongest exercise intentions, while individuals with low goal attainability and an external attribution style will demonstrate the weakest exercise intentions*.

Hypotheses for Experiment 2:

**Hypothesis 5a** **(H5a):** *The direction of the attribution style shift will significantly affect changes in health beliefs and exercise intentions. Individuals shifting from an external to an internal attribution style will exhibit a significant increase in exercise intentions*.

**Hypothesis 5b** **(H5b):** *The direction of the attribution style shift will significantly affect changes in health beliefs and exercise intentions. Individuals shifting from an internal to an external attribution style will exhibit a significant decrease in exercise intentions*.

**Hypothesis 6** **(H6):** *There is an interaction effect between goal attainability and the direction of attribution style change. If H6 is supported, further testing of H7 will proceed; if H6 is not supported, H7 will be discarded*.

**Hypothesis 7a** **(H7a):** *Under high goal attainability conditions, individuals shifting from an external to an internal attribution style will exhibit the highest health beliefs. Under low goal attainability conditions, individuals shifting from an internal to an external attribution style will exhibit the lowest health beliefs*.

**Hypothesis 7b** **(H7b):** *Under high goal attainability conditions, individuals shifting from an external to an internal attribution style will exhibit the highest exercise intentions. Under low goal attainability conditions, individuals shifting from an internal to an external attribution style will exhibit the lowest exercise intentions*.

## 3. Research Methods

### 3.1. Method of Experiment 1

#### 3.1.1. Research Design and Type for Experiment 1

Experiment 1 adopted a 2 (goal attainability: high vs. low) × 2 (attribution style: external vs. internal) between-subjects experimental design, resulting in four experimental groups. The objective was to explore the effects of goal attainability and attribution style on health beliefs and exercise intentions, as well as to analyze their interaction effects. Participants underwent a systematic evaluation through a pre-test, a post-manipulation test, and a post-experiment test to assess the impact of goal attainability and attribution style shifts on health beliefs and exercise intentions.

#### 3.1.2. Participants and Sampling Criteria for Experiment 1

Experiment 1 recruited 150 undergraduate students from universities in China using a convenience sampling approach. To ensure eligibility, participants were required to meet the following criteria: aged between 19 and 24 years and enrolled as full-time university students; with prior experience in regular physical activity; in good physical and mental health, with unimpaired cognitive and communicative functioning; and having signed a written informed consent form prior to participation. Exclusion criteria encompassed the following: individuals currently undergoing medical treatment or physical rehabilitation; those who had recently experienced major life events likely to influence their emotional or physical state; and any condition deemed likely to interfere with the validity of the experimental outcomes.

During the course of the experiment, four participants withdrew for personal reasons, resulting in a final sample of 146 individuals who completed the full procedure. Among them, 72 were male. All participants provided written informed consent prior to participation, and the study protocol adhered strictly to institutional ethical guidelines and approval procedures.

#### 3.1.3. Measurement Instruments for Experiment 1

**Figure 1:**
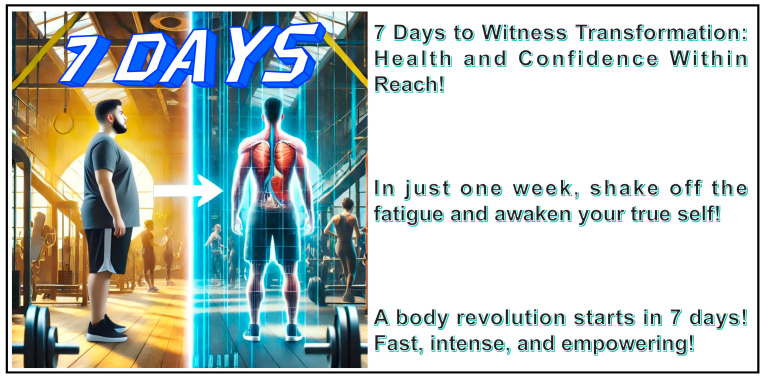
High goal accessibility advertisement image (1).

**Figure 2:**
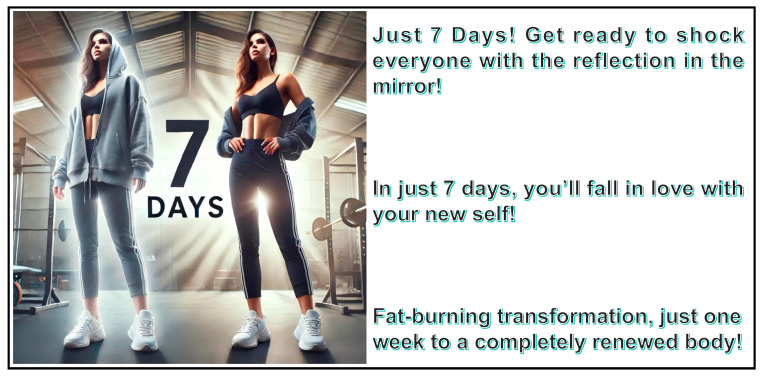
High goal accessibility advertisement image (2).

**Figure 3:**
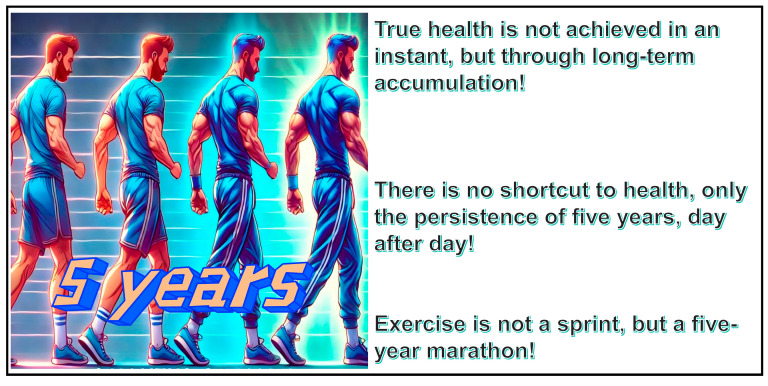
Low goal accessibility advertisement image (1).

**Figure 4:**
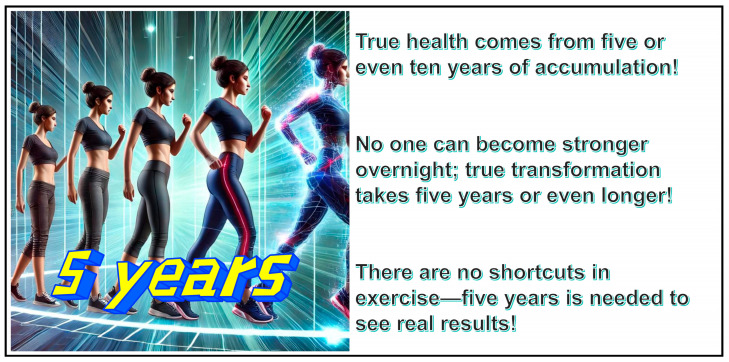
Low goal accessibility advertisement image (2).

In Experiment 1, image-based stimuli were used to manipulate the independent variables, and structured questionnaires were employed for measurement. To manipulate goal attainability, two sets of advertisement images were developed to elicit differential perceptions of goal difficulty. Participants in the high attainability condition viewed images (Figure 1 and Figure 2) emphasizing clear, actionable fitness goals with immediate or short-term benefits. In contrast, the low attainability condition featured abstract, distant messaging paired with blurred backgrounds or long-shot compositions (Figure 3 and Figure 4), designed to evoke a greater sense of psychological distance and perceived difficulty in goal achievement.

**Figure 5:**
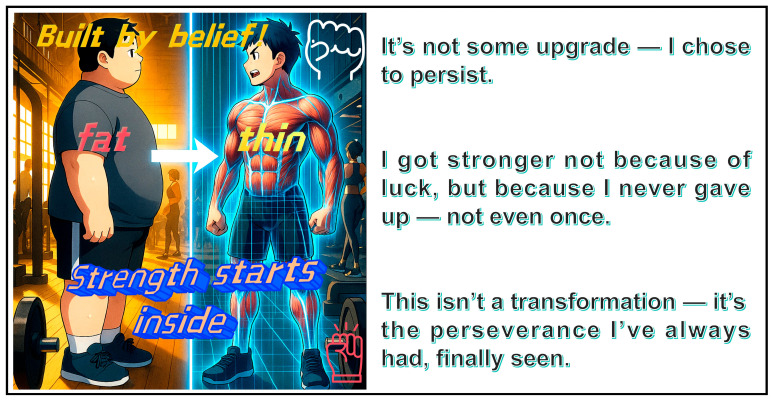
Advertisement image employing an internal attribution style (1).

**Figure 6:**
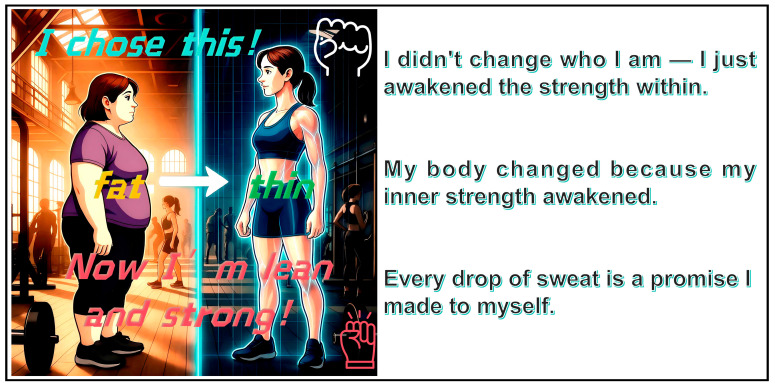
Advertisement image employing an internal attribution style (2).

**Figure 7:**
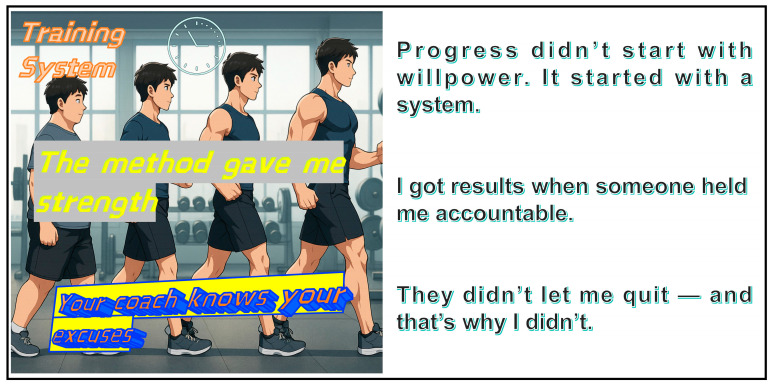
Advertisement image employing an external attribution style (1).

**Figure 8:**
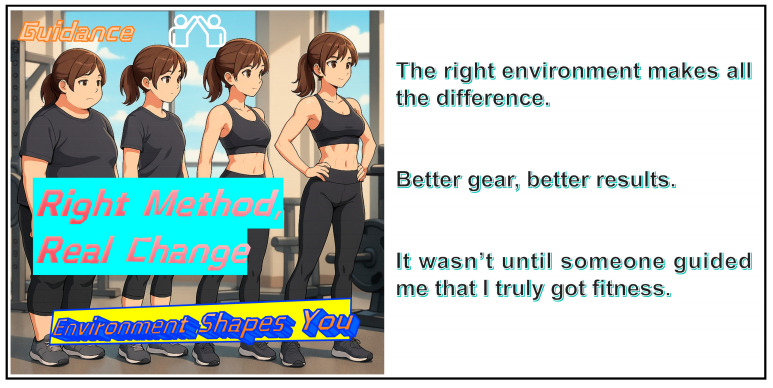
Advertisement image employing an external attribution style (2).

For the manipulation of attribution style, Experiment 1 also employed image-based intervention materials. The internal attribution condition featured images (Figure 5 and Figure 6) that emphasized individual willpower, self-discipline, and personal effort, accompanied by visual elements symbolizing intrinsic motivation and positive emotional tone. Conversely, the external attribution condition presented images (Figure 7 and Figure 8) that underscored the determining role of external circumstances, visually highlighting non-personal factors such as equipment, environmental conditions, or social influence.

The primary measuring tools used in Experiment 1 include the health belief scale, exercise intention scale, goal attainability perception scale, and attribution style scale. The health belief scale was adapted from the HBM and includes two items related to health behavior cognition: “I believe that exercise can significantly improve my health condition” and “Even without short-term effects, exercise is still important for my health” (Villar et al., 2017; Wu et al., 2020). A 7-point Likert scale was used for scoring, with 1 = Strongly Disagree and 7 = Strongly Agree.

The exercise intention scale was adapted from Teixeira et al.’s (2012) study to assess individuals’ willingness to engage in physical activity. It includes two items: “I plan to exercise at least three times per week in the future, if given the opportunity” and “I am willing to engage in more physical exercise”. The same 7-point Likert scale was used for scoring.

The goal attainability perception scale was adapted from Locke and Latham’s (2002) study to assess participants’ perceptions of the attainability of exercise goals. It includes items such as “I believe the effects of exercise can be quickly observed” and “I think exercise requires long-term commitment to see significant results”. The scale includes one positively worded item and one reverse-worded item, with score inversion applied during data analysis.

The attribution style scale was adapted from the dual-item brief scale developed based on Weiner’s Attribution Theory. It includes the items “My exercise outcomes primarily depend on my effort” and “External factors, such as environment, weather, and genetics, are more important than personal effort”. The scale uses a 7-point Likert scale for scoring (S. J. Biddle et al., 2001). The scale includes one positively worded item and one reverse-worded item, with score inversion applied during data analysis.

The full version of the scale used in Experiment 1 can be found in Appendix A.

#### 3.1.4. Experimental Procedure for Experiment 1

In Experiment 1, all participants initially completed a pre-test to measure health beliefs, exercise intentions, goal attainability perception, and attribution style. This pre-test ensured balanced baseline data across all groups before the experiment began and provided reliable foundational data for subsequent experimental manipulations. Participants were then evenly assigned to groups based on gender to ensure similar gender distribution across the experimental conditions. The experimental groups consisted of four conditions: Group A (high goal attainability + internal attribution), Group B (high goal attainability + external attribution), Group C (low goal attainability + internal attribution), and Group D (low goal attainability + external attribution).

After group assignment, participants were shown two high goal accessibility advertisement images (Images 1 and 2) and two low goal accessibility advertisement images (Images 3 and 4), with the content of these advertisements specifically tailored to align with the experimental manipulations for each group. Subsequently, participants viewed two internal attribution style advertisement images (Images 5 and 6) and two external attribution style advertisement images (Images 7 and 8) to influence their attribution style. To ensure gender balance in the experimental stimuli, each participant was exposed to one male-targeted and one female-targeted advertisement in every viewing round. This design minimizes potential gender-matching biases—such as the perception that the ad is intended exclusively for one’s own gender—and enhances both the ecological validity and generalizability of the intervention. Upon completing the advertisement viewing, participants filled out post-manipulation and post-test questionnaires, assessing health beliefs, exercise willingness, goal accessibility, and attribution style. The effectiveness of the experimental manipulations was then analyzed, marking the conclusion of Experiment 1. After the experiment, participants were debriefed and informed that the content of the advertisements was artificially created for the purposes of this study and did not represent actual research findings.

#### 3.1.5. Experimental Description for Experiment 1

Experiment 1 was designed to examine the main and interactive effects of goal attainability and attribution style on university students’ health beliefs and exercise intentions. Using a 2 (goal attainability: high vs. low) × 2 (attribution style: internal vs. external) between-subjects factorial design, the study investigated how variations in contextual cues influence cognitive and motivational responses.

Goal attainability was manipulated through exposure to exercise advertisement images depicting varying temporal expectations for health outcomes. In the high attainability condition, advertisements emphasized “visible positive changes in the short term”, whereas in the low attainability condition, they stressed “long-term persistence required for noticeable effects”.

Attribution style was similarly manipulated via image content. Participants in the internal attribution group viewed messages highlighting “the decisive role of personal effort and persistence”, while those in the external attribution group encountered content attributing outcomes to “uncontrollable external factors such as weather, environment, or genetics”.

The study involved no physical risk or real-world behavioral intervention; all stimuli were purposefully constructed to simulate psychologically realistic scenarios for the manipulation of perception and belief.

#### 3.1.6. Data Analysis Methods and Software for Experiment 1

All statistical analyses in Experiment 1 were conducted using IBM SPSS Statistics 23.0. Descriptive statistics, including means and standard deviations, were first calculated for the primary variables—health beliefs, exercise intention, perceived goal attainability, and attribution style—to provide an overview of group-level characteristics.

Prior to the main analyses, a one-way ANOVA was performed on pre-test scores to ensure that the four experimental groups did not differ significantly on baseline measures, thereby confirming initial equivalence. A chi-square test was also conducted to verify that gender distribution was balanced across conditions.

To assess the effectiveness of the experimental manipulations, paired samples *t*-tests were conducted on perceived goal attainability and attribution style measures, comparing pre- and post-manipulation scores to determine whether the manipulations produced the intended effects.

To formally test the hypotheses, independent samples *t*-tests were conducted to examine the main effects of goal attainability and attribution style on health beliefs and exercise intentions. In addition, a two-way ANOVA was performed to assess the significance of the interaction between goal attainability and attribution style.

### 3.2. Method of Experiment 2

#### 3.2.1. Research Design and Type for Experiment 2

Experiment 2 adopted a 2 (goal attainability: high vs. low) × 2 (attribution style shift: external → internal vs. internal → external) between-subjects experimental design, resulting in four experimental groups. The objective was to explore the effects of goal attainability and attribution style shift on health beliefs and exercise intentions, as well as to further analyze their interaction effects. Experiment 2 systematically assessed the impact of goal attainability and attribution style shift on health beliefs and exercise intentions through a design that included a pre-test, post-manipulation test, and post-experiment test.

#### 3.2.2. Participants and Sampling Criteria for Experiment 2

Experiment 2 employed a convenience sampling strategy to recruit 138 undergraduate students from universities in China, all of whom met the same inclusion and exclusion criteria established in Experiment 1. Eligibility was based on age range, prior experience with physical activity, good physical and mental health, and the provision of informed consent. During the course of the study, eight participants withdrew for personal reasons, resulting in a final sample of 130 students who completed the full experimental protocol. Participants were randomly assigned to experimental conditions as per the study design. The final sample comprised 64 males and 66 females, yielding a relatively balanced gender distribution.

All participants provided written informed consent prior to participation, and the study was conducted in strict accordance with institutional ethical guidelines and research ethics regulations.

#### 3.2.3. Measurement Instruments for Experiment 2

Experiment 2 employed multimodal stimulus materials and structured questionnaires to manipulate goal attainability and attribution style, and to measure associated psychological variables. The intervention incorporated both images and videos, with each type of material corresponding to one of the two manipulated variables. Goal attainability was manipulated using the same image-based materials as in Experiment 1—health-related exercise advertisements depicting either high or low goal attainability (Images 1–4)—to elicit different perceptions of task difficulty. Attribution style was manipulated through short video stimuli, each approximately two minutes in length, designed to reinforce either an internal or external attributional frame. The content of each video reflected the assigned attributional shift (internal-to-external vs. external-to-internal), ensuring consistency with the participant’s experimental condition. Participants in the internal-to-external attribution groups viewed a two-minute video emphasizing the influence of external conditions on athletic outcomes. The video featured a demonstrator in a white lab coat presenting a specific brand of athletic footwear. Through a series of live-action segments, the video objectively compared the featured shoes with standard models, showcasing differences in sole elasticity, impact absorption during foot strikes, and traction performance. These technical tests were paired with motion analyses of athletes performing the same exercises in different footwear, highlighting how external equipment can critically enhance athletic performance. The video adopted a neutral, analytical tone with no voiceover narration or emotional cues, relying solely on visual evidence and demonstrator-led demonstrations to convey an attributional message: external conditions play a decisive role in determining athletic success. Conversely, participants in the external-to-internal attribution groups viewed a two-minute video designed to highlight the primacy of personal effort in achieving athletic success. Featuring several young athletes (both male and female), the footage documented authentic training scenarios in which individuals pushed their physical limits through endurance runs, high-intensity interval training, and strength workouts. Shot in a documentary style and accompanied by rhythmically intense, motivational background music, the video conveyed the sweat, struggle, and persistence involved in overcoming personal barriers. Through powerful visual and auditory cues, the video reinforced an internal attribution frame—namely, that success is earned through sustained effort—thus promoting a self-directed view of achievement.

The measurement instruments used in Experiment 2 were identical to those employed in Experiment 1, including four adapted scales assessing health beliefs, exercise intention, perceived goal attainability, and attribution style (see Appendix A for full scale details). To evaluate the effectiveness of the experimental manipulations, goal attainability and attribution style were measured both before and after the intervention. Health beliefs and exercise intention, serving as the primary dependent variables, were assessed at pre- and post-test to capture changes in cognitive and motivational responses.

#### 3.2.4. Experimental Procedure for Experiment 2

In Experiment 2, all participants first completed a pre-test to measure health beliefs, exercise intentions, goal attainability perception, and attribution style. This ensured baseline data balance across the groups before the experiment began and provided reliable foundational data for subsequent experimental manipulations. Next, participants were categorized based on their attribution style using a dual judgment criterion and were evenly distributed according to gender to ensure a balanced gender ratio across the experimental groups. The experimental groups consisted of four conditions: Group A (High Goal Attainability + Internal Attribution → External Attribution), Group B (High Goal Attainability + External Attribution → Internal Attribution), Group C (Low Goal Attainability + Internal Attribution → External Attribution), and Group D (Low Goal Attainability + External Attribution → Internal Attribution).

After the group assignment, participants first viewed exercise advertisements related to goal attainability to manipulate their perceptions of goal attainability. Participants in the high goal attainability groups (Groups A and B) watched advertisements emphasizing the ease of achieving exercise goals (Images 1 and 2, consistent with Experiment 1), while participants in the low goal attainability groups (Groups C and D) watched advertisements highlighting the need for long-term effort to achieve exercise goals (Images 3 and 4, consistent with Experiment 1).

Next, participants viewed the video corresponding to their assigned attributional shift condition. Those in the internal-to-external attribution groups (Groups A and C) watched the external attribution video, while those in the external-to-internal attribution groups (Groups B and D) watched the internal attribution video. After watching the videos, participants completed measurement questionnaires assessing health beliefs, exercise intentions, goal attainability, and attribution style, followed by post-manipulation and post-experiment tests. At this point, Experiment 2 was concluded. After the experiment, participants were debriefed and informed that all the experimental content, including the advertisements and videos, was specifically designed for this study and did not represent actual research findings.

#### 3.2.5. Experimental Description for Experiment 2

Experiment 2 aimed to further examine the effects of goal attainability and attributional shift (i.e., transformation of attribution style) on health beliefs and exercise intention, with a particular focus on their interaction mechanism. In contrast to Experiment 1, which manipulated static attribution styles, this study introduced a dynamic transformation of attribution by guiding participants—via tailored video stimuli—to shift from internal to external attribution or vice versa, thereby simulating attributional changes as they might occur in real-world contexts.

Goal attainability was manipulated using exercise-related image advertisements, as in Experiment 1. Attribution transformation, however, was operationalized through customized video interventions designed to induce directional shifts in attributional reasoning. The experiment adopted a 2 (goal attainability: high vs. low) × 2 (attributional shift: internal → external vs. external → internal) between-subjects factorial design. By incorporating multimodal stimuli, the study sought to enhance ecological validity and to explore the interactive effects between attributional transformation and perceived goal attainability.

#### 3.2.6. Data Analysis Methods and Software for Experiment 2

All statistical analyses in Experiment 2 were conducted using IBM SPSS Statistics 23.0. In addition to replicating the analytic procedures used in Experiment 1—including descriptive statistics, chi-square tests, one-way ANOVAs, paired samples *t*-tests, independent samples *t*-tests, and two-way ANOVAs—this study incorporated extended analyses tailored to the specific features of the experimental design.

Prior to group assignment, participants’ baseline attribution styles were classified using a dual-criterion pre-screening method: (1) a predefined cutoff score on the internal and external attribution subscales, and (2) a calculated attribution bias index, defined as the difference between internal and external scores. To ensure the validity of subsequent experimental manipulation, only participants exhibiting distinct attribution tendencies were included in the final sample.

To formally test the hypotheses, independent samples *t*-tests were conducted to examine the main effects of attributional shift direction (internal → external vs. external → internal) on health beliefs and exercise intention. In addition, a two-way ANOVA was performed to assess whether there was a significant interaction between goal attainability and attributional shift direction.

## 4. Experimental Results

### 4.1. Results of Experiment 1

#### 4.1.1. Pre-Test for Experiment 1

Prior to the formal experiment, a pre-test was conducted to verify the baseline equivalence of the experimental groups in terms of health beliefs, exercise intentions, goal attainability, and attribution style. The pre-test helped ensure that there were no significant initial differences between the groups, thus preventing individual differences from influencing the effectiveness of the experimental manipulations. Additionally, the pre-test was used to assess whether any interaction between goal attainability and attribution style already existed before the experiment began, ensuring the validity of the experimental manipulation.

As shown in Table 1, this study employed a gender-balanced allocation strategy during the group assignment process to control for potential gender effects on the research outcomes. A chi-square test (χ^2^ test) was conducted to examine whether the gender distribution across experimental groups was balanced. The results revealed no significant difference in the gender distribution among the experimental groups, χ^2^(3) = 0.159, *p* = 0.984 (*p* > 0.05), indicating that there was no systematic bias in the gender distribution across groups. These results indicate that the gender-balanced allocation strategy was successfully implemented in this study, minimizing the potential influence of gender on the experimental outcomes and thereby ensuring the reliability of the study’s internal validity.

In the pre-test, all variables were measured using a 7-point Likert scale (1 = Strongly Disagree, 7 = Strongly Agree). Some measurement items were reverse-worded, and reverse scoring was applied during data collection and analysis to ensure the accuracy and consistency of the measurement data. Specific details are provided in Table 2.

To assess whether there were significant differences in the variables across the experimental groups prior to the experiment, a one-way Analysis of Variance (ANOVA) was conducted. The pre-test results showed that the *p*-values for all variables were greater than 0.05 (Health Beliefs: F = 1.043, *p* = 0.376; Exercise Willingness: F = 1.323, *p* = 0.269; Goal Attainability: F = 2.586, *p* = 0.056; Attribution Style: F = 0.874, *p* = 0.456), indicating that there were no significant differences among the experimental groups in terms of health beliefs, exercise intentions, goal attainability, and attribution style. This finding further validates the balance of the experimental groups and provides reliable baseline data for subsequent experimental manipulations.

To examine whether there was an interaction between goal attainability and attribution style on health beliefs and exercise intentions prior to the experiment, a two-way Analysis of Variance (ANOVA) was conducted. The pre-test results showed that the interaction between goal attainability and attribution style was not significant for both health beliefs and exercise intentions, with *p*-values greater than 0.05 (0.353 and 0.218, respectively). Despite relatively high partial eta squared values (0.512 and 0.538), the lack of statistical significance in the *p*-values means that no interaction effect between the two variables was observed before the experiment. Therefore, it can be concluded that there was no significant interaction between goal attainability and attribution style prior to the experiment.

#### 4.1.2. Post-Manipulation Test for Experiment 1

The purpose of the post-manipulation test was to verify whether the experimental manipulations were successful, specifically assessing whether goal attainability and attribution style changed in the expected direction across the groups after viewing the exercise advertisements. To assess the success of the experimental manipulations for goal attainability and attribution style, participants completed measurements of these variables both before and after the experiment. Paired samples *t*-tests were conducted to compare the pre- and post-manipulation data, determining whether the experimental manipulation significantly altered participants’ perceptions of goal attainability and attribution style. Table 3 presents the means, standard deviations, and statistical test results for each experimental group before and after the manipulation.

In terms of goal attainability manipulation, the results of the paired samples *t*-test showed that, compared to the pre-test, participants in the high goal attainability groups (Groups A and B) exhibited a significant increase in goal attainability perception in the post-test, while participants in the low goal attainability groups (Groups C and D) showed a significant decrease in their goal attainability perception. These results indicate that the experimental manipulation successfully guided participants’ differing perceptions of goal attainability. Specifically, participants in the high goal attainability groups were more likely to perceive exercise goals as more achievable, while participants in the low goal attainability groups were more inclined to perceive exercise goals as more difficult to attain, consistent with the experimental design’s expectations.

Similarly, in terms of attribution style manipulation, the results showed that, compared to the pre-test, participants in the high attribution groups (Groups A and C) exhibited a significant increase in their attribution style scores in the post-test, while participants in the low attribution groups (Groups B and D) showed a significant decrease in their attribution style scores. These results indicate that the experiment successfully guided participants’ attributional reasoning regarding exercise outcomes. Specifically, participants in the high attribution groups were more likely to believe that exercise results were determined by personal effort, while participants in the low attribution groups were more likely to believe that exercise outcomes were influenced by external environmental factors, consistent with the experimental expectations.

#### 4.1.3. The Effect of Goal Attainability on Health Beliefs and Exercise Intentions (Testing H1)

Table 4 presents the results of the independent samples *t*-test for the effect of goal attainability on health beliefs and exercise intentions. The analysis revealed that participants in the high goal attainability group (M = 4.80, SD = 1.55) had significantly higher health belief scores compared to those in the low goal attainability group (M = 3.81, SD = 1.36), with t(144) = 4.11, *p* < 0.001. The mean difference was 0.99, with a 95% confidence interval [0.51, 1.46]. This result supports Hypothesis H1, indicating that higher goal attainability is associated with stronger health beliefs.

In terms of exercise willingness, due to unequal variances (Levene’s test for equality of variances, *p* < 0.05), a *t*-test assuming unequal variances was conducted. The results revealed that participants in the high goal attainability group (M = 4.85, SD = 1.12) had significantly higher exercise willingness scores compared to those in the low goal attainability group (M = 4.42, SD = 1.48), with t(142.36) = 1.98, *p* = 0.049. The 95% confidence interval was [0.001, 0.85]. This indicates that the effect of goal attainability on exercise willingness reached a significant level, suggesting that higher goal attainability is associated with stronger exercise intentions.

Taken together, the results support Hypothesis H1. Compared to participants in the low goal attainability condition, those in the high attainability group reported significantly higher levels of health beliefs and exercise intention. These findings suggest that greater perceived goal attainability facilitates stronger health-related cognition and behavioral motivation, aligning with the directional predictions of the hypothesis.

#### 4.1.4. The Effect of Attribution Style on Health Beliefs and Exercise Intentions (Testing H2)

Table 5 presents the results of the independent samples *t*-test for the effect of attribution style on health beliefs and exercise intentions. The analysis revealed that individuals with an internal attribution style had significantly higher health belief scores compared to those with an external attribution style, with t(144) = 2.04, *p* = 0.044. The mean difference was 0.51, with a 95% confidence interval [0.01, 1.00]. This indicates that individuals who are more likely to attribute exercise outcomes to their own effort tend to have stronger health beliefs, supporting Hypothesis H2.

In terms of exercise willingness, due to unequal variances (Levene’s test for equality of variances, *p* < 0.05), a *t*-test assuming unequal variances was conducted. The results revealed that participants in the internal attribution group (M = 5.04, SD = 1.15) exhibited significantly higher exercise willingness scores compared to those in the external attribution group (M = 4.24, SD = 1.39), with t(142.22) = 3.79, *p* < 0.001. The 95% confidence interval was [0.38, 1.22]. This further supports Hypothesis H2, indicating that attribution style has a significant impact on exercise willingness, with individuals who attribute exercise outcomes to their own effort showing a stronger intention to engage in physical activity than those who attribute outcomes to external factors.

Taken together, the results provide strong support for Hypothesis H2. Compared to individuals adopting an external attribution style, those employing an internal attribution style exhibited significantly higher scores in both health beliefs and exercise intention. These findings suggest that attribution style plays a decisive role in shaping Chinese college students’ health-related cognition and motivation, with internal attributions more conducive to fostering positive beliefs and intentions toward health-promoting behaviors.

#### 4.1.5. Interaction Analysis of Goal Attainability and Attribution Style (Testing H3)

To examine whether there is a significant interaction between goal attainability and attribution style on health beliefs and exercise intentions, a two-way Analysis of Variance (ANOVA) was conducted for statistical testing.

Table 6 presents the results of the between-subjects effects test for goal attainability and attribution style on health beliefs. The interaction term of goal attainability × attribution style yielded an F-value of 1.138, with *p* = 0.307 (*p* > 0.05), indicating that the interaction effect did not reach significance (η^2^ = 0.503). This suggests that there was no significant interaction between goal attainability and attribution style on health beliefs, meaning that health belief scores did not show significant changes under different combinations of goal attainability and attribution style. As Hypothesis H3 was not supported in terms of health beliefs, there is no need to further test Hypothesis H4a.

Table 6 also presents the results of the between-subjects effects test for goal attainability and attribution style on exercise intentions. The interaction term of goal attainability × attribution style yielded an F-value of 1.531, with *p* = 0.049 (*p* < 0.05), indicating that the interaction effect reached significance (η^2^ = 0.577). This suggests that there was a significant interaction between goal attainability and attribution style on exercise intentions, meaning that the different combinations of goal attainability and attribution style had a significant impact on individuals’ exercise intentions. As Hypothesis H3 was supported for exercise intentions, Hypothesis H4b was further tested.

In summary, Hypothesis H3 was partially supported. The interaction between goal attainability and attribution style did not reach statistical significance for health beliefs, indicating that the combination of these two factors did not significantly influence individuals’ cognitive evaluations of health. Therefore, H3 was not supported in the domain of health beliefs. However, the interaction effect on exercise intention was statistically significant, suggesting that different combinations of goal attainability and attribution style produced meaningful variations in participants’ willingness to engage in physical activity. These findings support H3 in the dimension of exercise intention and serve as the basis for further examination of Hypothesis H4b.

#### 4.1.6. The Effects of Goal Attainability and Attribution Style on Exercise Intentions (Testing H4b)

Building on the validation of Hypothesis H3 (the significant interaction between goal attainability and attribution style on exercise intentions, *p* = 0.049), this section further analyzes the changes in exercise intentions across the different experimental groups to test Hypothesis H4b: Individuals with high goal attainability and an internal attribution style will have the strongest exercise intentions, while individuals with low goal attainability and an external attribution style will have the weakest exercise intentions.

Table 7 shows the effect of goal attainability and attribution style on exercise intentions (pre-test vs. post-test). The high goal attainability + internal attribution group (Group A) exhibited the largest increase in exercise intentions (ΔM = +1.20), indicating that under the perception of more achievable exercise goals, individuals are more likely to attribute exercise outcomes to their own effort, thereby enhancing their exercise intentions.

The high goal attainability + external attribution group (Group B) showed a notable increase in exercise intentions (ΔM = +0.93), although it was lower than Group A. This suggests that while high goal attainability contributes to enhancing exercise intentions, if individuals attribute exercise outcomes to external factors, their exercise motivation remains relatively lower.

The low goal attainability + internal attribution group (Group C) exhibited a smaller increase in exercise intentions (ΔM = +0.68), indicating that even though individuals believe their effort is crucial to exercise outcomes, if they perceive exercise goals as difficult to attain, the growth in their exercise intentions remains limited.

The low goal attainability + external attribution group (Group D) showed a decrease in exercise intentions (ΔM = −0.43), indicating that when individuals perceive exercise goals as difficult to achieve and attribute outcomes to external factors, their exercise intentions are weakest and may even decrease.

In summary, Hypothesis H4b is supported, indicating that the different combinations of goal attainability and attribution style significantly affect changes in individuals’ exercise intentions.

### 4.2. Results of Experiment 2

#### 4.2.1. Pre-Test for Experiment 2

Experiment 2 employed a 2 (goal attainability: high vs. low) × 2 (attribution style shift: external to internal vs. internal to external) between-subjects experimental design, resulting in four experimental groups. Participants first completed the pre-test, followed by random assignment to one of the four experimental groups, where they underwent different manipulations of goal attainability and attribution style shift. Finally, participants completed the post-manipulation and post-experiment tests.

To ensure the effectiveness of the attribution style shift manipulation and the controllability of its psychological basis, this study systematically assessed participants’ initial attribution tendencies prior to the formal intervention in Experiment 2. The attribution style measurement tool used was consistent with that in Experiment 1, consisting of a dual-item brief scale based on Attribution Theory, designed to identify an individual’s primary attribution direction based on the principle of minimal interference. Although the tool contains only two items, its construct is clear, and it has been validated in Experiment 1 through a large-sample pre-test, demonstrating good discriminative ability and experimental validity. It is suitable for classification and manipulation in short-duration experimental settings. To further enhance the sensitivity of attribution tendency identification and the validity of the grouping, the study employed a dual judgment criterion for classification:

The first criterion was that if participants scored higher than 5 on the internal attribution items and lower than 4 on the external attribution items, they were classified as “initial internal attribution users”. Conversely, if participants scored higher than 5 on the external attribution items and lower than 4 on the internal attribution items, they were classified as “initial external attribution users”.

The second criterion involved calculating the “Attribution Bias Index” (the internal attribution score minus the external attribution score) to assess the consistency and significance of participants’ attribution orientation. If the absolute value of the bias index was ≥1, participants were classified as having a clear attribution tendency. If the bias index was between −1 and +1, participants were classified as having an unclear attribution tendency and were excluded to avoid interfering with the purity of the manipulation effect.

Participants who met “Criterion 1” (internal attribution > 5 and external attribution < 4, or vice versa) or had an absolute value of the “Attribution Bias Index” ≥ 1 were considered to have a clear attribution orientation and were included in the formal experimental procedure. Through the dual judgment mechanism, it was ensured that each participant had a clear attribution orientation before the experiment began, providing the necessary psychological foundation and grouping criteria for subsequent attribution style shift manipulations (internal attribution shift or external attribution shift).

As shown in Table 8, this study employed a gender-balanced allocation strategy during the group assignment process to control for potential gender effects on the research outcomes. A chi-square test (χ^2^ test) was used to assess whether the gender distribution was balanced across the experimental groups. The results revealed no significant differences in gender distribution across the groups, χ^2^(3) = 0.032, *p* = 0.999 (*p* > 0.05), indicating that there was no systematic bias in the gender distribution between the experimental groups. This finding demonstrates the successful implementation of the gender-balanced allocation strategy, ensuring that gender factors did not have a potential impact on the experimental outcomes, thereby confirming the reliability of the internal validity of the experiment.

In the pre-test, all variables were measured using a 7-point Likert scale (1 = Strongly Disagree, 7 = Strongly Agree). Some of the measurement items were reverse-coded, and reverse scoring was applied during data collection and analysis to ensure the accuracy and consistency of the measurement data. Specific details for each group are shown in Table 9.

To examine whether there were significant differences in the variables across the experimental groups before the experiment, a one-way analysis of variance (ANOVA) was conducted, with specific results shown in Table 10. The pre-test results revealed that the *p*-values for all variables were greater than 0.05, indicating no significant differences between the experimental groups in terms of health beliefs, exercise intentions, goal attainability, and attribution style. This further validates the balance of the experimental groups, providing reliable baseline data for subsequent experimental manipulations. Additionally, the results from Experiment 1 have already demonstrated whether an interaction exists between goal attainability and attribution style on health beliefs and exercise intentions. Therefore, no interaction effect measurement was conducted in the pre-test of Experiment 2.

#### 4.2.2. Post-Manipulation Test for Experiment 2

The purpose of the post-manipulation test is to assess whether the experimental manipulation was successful, specifically by examining whether participants’ attributional styles in each group have shifted in accordance with the experimental design after viewing the sports advertisements and videos. To validate the effectiveness of the manipulation of goal accessibility and attributional styles, participants completed measurements of these variables both before and after the experiment. Paired samples *t*-tests were conducted to compare pre- and post-manipulation data, determining whether the experimental manipulation significantly altered participants’ perceptions of goal accessibility and attributional styles. Table 11 presents the means, standard deviations, and statistical results for each experimental group before and after manipulation. Statistical analysis confirmed that the manipulation successfully altered goal accessibility and attributional styles, thereby validating the experimental design.

#### 4.2.3. The Impact of Attribution Style Transformation on Health Beliefs and Exercise Willingness (Testing H5)

To examine the effect of the direction of attribution style transformation on individuals’ health beliefs and exercise willingness, an independent sample *t*-test was conducted to compare individuals who shifted from internal attribution to external attribution (referred to as the “Internal → External group”) and those who shifted from external attribution to internal attribution (referred to as the “External → Internal group”). The results, presented in Table 12, show that significant differences in both health beliefs and exercise willingness were found between the two groups (Health Beliefs: t = −6.460, *p* < 0.001; Exercise Willingness: t = −6.030, *p* < 0.001). The mean differences were −1.547 and −1.436, respectively, and the 95% confidence intervals did not include zero, indicating that the differences were statistically significant. These findings validate Hypotheses H5a and H5b, confirming that attribution style transformation significantly affects individuals’ psychological variables related to exercise.

#### 4.2.4. Interaction Analysis of Goal Accessibility and Attribution Style Transformation (Testing H6)

To examine whether there is a significant interaction between goal accessibility and attribution style transformation in relation to health beliefs and exercise willingness, a two-way Analysis of Variance (two-way ANOVA) was conducted. According to the experimental results (see Table 13), for health beliefs, the interaction F-value was 0.816, *p* = 0.772, and partial Eta squared was 0.496. These results indicate that the interaction between goal accessibility and attribution style did not reach a significant level in relation to changes in health beliefs, failing to support Hypothesis H6 in the domain of health beliefs. Therefore, further testing of H7a is unnecessary.

However, the analysis for exercise willingness revealed a significant interaction effect. The interaction F-value was 1.691, *p* = 0.031, and partial Eta squared was 0.671, indicating that the interaction between goal accessibility and attribution style transformation significantly affected exercise willingness, supporting the validity of Hypothesis H6 in relation to exercise willingness. Based on this result, further testing of H7b will be conducted.

#### 4.2.5. The Impact of Goal Accessibility and Attribution Style Transformation on Exercise Willingness (Testing H7b)

Building on the validation of H6, this section examines the specific impact of attribution style transformation on exercise willingness under different goal accessibility conditions. According to the results in Table 14, Group B exhibited a significant increase in exercise willingness from 4.000 ± 1.369 at pre-test to 5.424 ± 1.024 at post-test, with a change of +1.424, marking the largest increase in exercise willingness. In contrast, Group C’s exercise willingness decreased significantly from 4.438 ± 1.268 at pre-test to 3.047 ± 1.187 at post-test, with a change of −1.391, showing the smallest change in exercise willingness. Therefore, Hypothesis H7b is supported, confirming that under low goal accessibility conditions, individuals who transformed from internal attribution to external attribution exhibited the lowest exercise willingness, while under high goal accessibility conditions, individuals who transformed from external attribution to internal attribution exhibited the highest exercise willingness. This finding suggests that both goal accessibility and attribution style transformation play a significant role in changes in individual exercise willingness. High goal accessibility enhances exercise willingness in individuals who shift from external to internal attribution, while low goal accessibility suppresses exercise willingness in individuals who shift from internal to external attribution.

## 5. Discussion

### 5.1. Discussion of Findings from Experiment 1

Experiment 1 explored the effects of goal attainability and attribution style on health beliefs and exercise intentions. The results indicate that both goal attainability and attribution style significantly influence individuals’ health beliefs and exercise intentions independently, with an additional interaction effect observed on exercise intentions. This finding provides new empirical support for the HBM and Attribution Theory, while also offering important insights for practical exercise motivation strategies.

Regarding Hypothesis 1 (H1), which posited that higher goal attainability would be associated with stronger health beliefs and exercise intentions, the results of Experiment 1 provided full support for this assumption. Individuals with high goal attainability exhibited significantly higher health beliefs and exercise intentions compared to those with low goal attainability. This suggests that an individual’s perception of the attainability of exercise goals is a key determinant of their exercise intentions. This result aligns with the concept of “perceived barriers” in the HBM, which posits that individuals are more likely to engage in a health behavior when they perceive it as easy to implement (Yan et al., 2024). In terms of exercise intentions, individuals who perceive exercise as yielding quick results and easy to maintain are more likely to develop positive health beliefs and higher exercise intentions (Seeman & Crimmins, 2001). Experiment 1 further confirms this through experimental manipulation, showing that different exercise advertisement messages successfully influenced individuals’ perceptions of goal attainability, leading to differences in their exercise intentions.

Regarding Hypothesis 2 (H2), which predicted that individuals with an internal attribution style would report stronger health beliefs and exercise intentions than those with an external attribution style, the findings fully supported this hypothesis. Regarding the impact of attribution style, Experiment 1 found that individuals with an internal attribution style exhibited significantly higher health beliefs and exercise intentions compared to those with an external attribution style. This suggests that individuals who attribute exercise outcomes to their own efforts are more likely to engage in exercise. This finding supports Weiner’s Attribution Theory, which posits that when individuals perceive success as controllable (e.g., achieving better exercise results through effort), they are more likely to persist in the behavior (Kavanagh et al., 2021). In contrast, if individuals believe that exercise outcomes are primarily influenced by external factors such as the environment, weather, or genetics, they may lack sufficient motivation to exercise (Alrasheeday et al., 2024). Experiment 1, through the experimental manipulation of attribution style, successfully altered individuals’ attributional reasoning about exercise outcomes after the advertisement intervention, thereby affecting their exercise intentions. This finding has important implications for exercise promotion strategies, suggesting that emphasizing individuals’ sense of control over exercise outcomes may be a key factor in enhancing the sustainability of exercise intentions.

Regarding Hypotheses 3, 4a, and 4b, the results partially supported H3: a significant interaction effect was found on exercise intentions, but not on health beliefs. As a result, Hypothesis 4a was not tested further, while Hypothesis 4b was fully supported—individuals in the high goal attainability × internal attribution group reported the highest exercise intentions. Regarding the interaction between goal attainability and attribution style, the results indicated that the interaction effect on health beliefs was not significant (*p* = 0.307), while the interaction effect on exercise intentions was significant (*p* = 0.049). This suggests that although goal attainability and attribution style independently influence health beliefs, their combination does not further amplify or weaken the formation of health beliefs. However, in terms of exercise intentions, individuals with high goal attainability and an internal attribution style showed the strongest exercise intentions, while those with low goal attainability and an external attribution style exhibited the weakest exercise intentions. This finding highlights the complexity of exercise motivation: when individuals perceive exercise goals as easy to achieve and attribute outcomes to their own effort, their motivation to exercise is at its highest; conversely, if they believe exercise is difficult to sustain and attribute outcomes to external factors, their likelihood of engaging in exercise may decrease, potentially leading to exercise avoidance behavior (Luszczynska, 2020). This result aligns with Social Cognitive Theory, which posits that an individual’s self-efficacy and sense of control over the external environment jointly determine their behavioral choices.

In addition, Experiment 1 sought to achieve a balanced presentation of gender information in the design of advertisement stimuli; all participants were exposed to images featuring both male and female figures during the viewing phase, thereby enhancing the ecological validity of the experimental procedure. However, it is important to note that the stimuli were not gender-matched to the participants; regardless of their own gender, all participants were exposed to mixed-gender imagery. This non-gender-matched design may have introduced a sense of perceived psychological distance during the processing of attributional information, potentially attenuating motivational arousal and reducing the cognitive weight assigned to judgmental cues. According to Social Identity Theory, individuals tend to project themselves onto and respond with greater empathy toward others who share similar personal characteristics. This effect is particularly evident in attributional judgments concerning self-efficacy and perceived control, where gender congruence may amplify the subjective relevance and behavioral implications of the information. Therefore, the effectiveness of the attributional manipulation in Experiment 1 may have been passively moderated by gender incongruence. Future research could incorporate gender-matched conditions to further investigate the interactive mechanisms between attributional framing and cognitive engagement.

### 5.2. Discussion of Findings from Experiment 2

Experiment 2 explored the effects of goal accessibility and attribution style transformation on individuals’ health beliefs and exercise willingness. The experimental results indicate that both goal accessibility and attribution style transformation not only have independent effects on health beliefs and exercise willingness, but also exhibit an interaction effect on exercise willingness. This finding provides new empirical support for the HBM and Attribution Theory, offering valuable insights for the development of effective exercise motivation strategies.

Regarding Hypotheses 5a and 5b, the results fully supported both: individuals who shifted from external to internal attribution exhibited significantly stronger health beliefs and exercise willingness, while those who shifted from internal to external attribution showed significant declines. Regarding the impact of attribution style, the experimental results showed that individuals with internal attribution scores were significantly higher in both health beliefs and exercise willingness compared to those with external attribution. This finding supports the hypothesis of Attribution Theory, which suggests that individuals who attribute their exercise outcomes to personal effort are more likely to engage in sustained exercise and maintain higher levels of exercise willingness. In contrast, individuals who believe that exercise outcomes primarily depend on external factors (such as the environment, weather, or genetics) may lack the motivation to continue exercising (S. Biddle et al., 2003). Experiment 2 experimentally manipulated participants’ attribution styles through advertising, influencing their exercise willingness. This finding provides important implications for exercise intervention practices, emphasizing that an individual’s sense of control over exercise outcomes may be key to enhancing exercise participation and long-term adherence (McAuley et al., 2011).

Regarding Hypotheses 6, 7a, and 7b, the results partially supported H6: a significant interaction effect was found on exercise willingness, but not on health beliefs. As a result, H7a was not supported, while H7b was confirmed—individuals in the high goal attainability × external-to-internal attribution group exhibited the greatest increase in exercise willingness. Regarding the interaction between goal accessibility and attribution style transformation, the results showed that their interaction on health beliefs was not significant (*p* = 0.307), but their interaction on exercise willingness was significant (*p* = 0.049). This finding suggests that while goal accessibility and attribution style independently affect health beliefs, their combination did not have a noticeable interactive effect on the formation of health beliefs. However, for exercise willingness, the interaction was significant; specifically, individuals with high goal accessibility and internal attribution exhibited the strongest exercise willingness, while individuals with low goal accessibility and external attribution exhibited the weakest exercise willingness. This result reveals the complexity of exercise willingness motivation: when individuals perceive exercise goals as achievable and attribute exercise outcomes to personal effort, their exercise willingness is strongest; conversely, when they perceive exercise goals as difficult to achieve and attribute outcomes to external factors, their exercise willingness is weakest, potentially even leading to exercise avoidance behavior (Bogg & Roberts, 2004). This finding is consistent with the concepts of self-efficacy and perceived environmental control in Social Cognitive Theory, suggesting that an individual’s exercise motivation is not only influenced by their perception of goal attainability but also by the strong influence of how they attribute exercise outcomes (Schunk & DiBenedetto, 2020).

In summary, Experiment 2 verified the independent effects of goal accessibility and attribution style transformation on health beliefs and exercise willingness, and revealed their interaction effect on exercise willingness. This provides a new perspective for exercise willingness research and offers a theoretical foundation for the design of exercise intervention strategies. Specifically, during intervention, enhancing individuals’ perception of goal accessibility and guiding them through appropriate attribution styles can effectively boost their exercise motivation, encouraging greater participation in physical exercise.

It is important to underscore that although gender was evenly distributed across groups in Experiment 2, the moderating role of gender on the effects of attributional shift interventions—namely, transitions from external to internal attributions and vice versa—was not further examined. Psychological mechanisms underlying attributional receptivity, motivational activation, and intention formation may differ by gender. For example, prior research suggests that women may exhibit stronger intrinsic motivation when self-regulatory messages are embedded in empathic, contextually resonant narratives, whereas men may be more sensitive to changes in perceived control over task outcomes. These findings imply that the effectiveness of attributional reorientation in Experiment 2 may have been subtly moderated by gender-specific cognitive–affective pathways. The absence of gender-based interaction analysis represents a key limitation of the present study. Future research should consider incorporating gender × intervention design frameworks or applying stratified statistical modeling to identify heterogeneity in responsiveness, thereby enhancing the precision, adaptability, and translational value of attribution-based health interventions.

### 5.3. Integrated Discussion

This research explored the effects of goal accessibility and attribution style on health beliefs and exercise willingness. The results showed that both goal accessibility and attribution style significantly influenced individuals’ health beliefs and exercise willingness independently, and also exhibited an interaction effect on exercise willingness. Through the design and analysis of Experiment 1 and Experiment 2, this research provides new empirical support for the HBM and Attribution Theory, while also offering important insights for the design of exercise intervention strategies.

Experiment 1 verified the significant impact of goal accessibility on health beliefs and exercise willingness. Individuals with high goal accessibility exhibited significantly higher health beliefs and exercise willingness compared to those with low goal accessibility. This finding supports the concept of “perceived barriers” in the Health Belief Model (Champion & Skinner, 2008), which suggests that individuals are more likely to engage in behaviors they perceive as easily achievable. Specifically, regarding exercise willingness, individuals who believe that the effects of exercise can be quickly achieved or are relatively easy to attain are more likely to develop positive health beliefs and demonstrate stronger exercise willingness. This aligns with the assumptions of perceived benefits, perceived barriers, and self-efficacy in the HBM (Janz & Becker, 1984). Furthermore, Experiment 1 successfully manipulated participants’ perceptions of goal accessibility through the use of exercise advertisements, influencing their exercise willingness, and confirmed that high goal accessibility effectively enhances individuals’ health beliefs and willingness to participate in exercise.

Experiment 1 found that attribution style also has a significant impact on health beliefs and exercise willingness. Individuals with internal attribution exhibited significantly higher health beliefs and exercise willingness compared to those with external attribution. This result supports the hypothesis of Attribution Theory, which suggests that individuals who attribute success to personal effort typically exhibit higher intrinsic motivation and sustained behavior. When individuals believe that exercise outcomes depend on their personal effort, they are more likely to maintain a high level of exercise willingness. In contrast, if individuals believe that outcomes are influenced by external factors (such as weather or genetics), they may lack sufficient motivation to persist in exercise. Therefore, exercise promotion strategies should focus on enhancing individuals’ sense of control and self-efficacy (Stuifbergen et al., 2010). Experiment 1 successfully manipulated participants’ attribution styles through advertising interventions, which significantly affected their exercise willingness, further validating the role of attribution style in promoting exercise participation.

Regarding the interaction between goal accessibility and attribution style, the results of Experiment 1 showed that their interaction on health beliefs was not significant (*p* = 0.307), but a significant interaction effect was found on exercise willingness (*p* = 0.049). This finding highlights the complexity of exercise willingness, suggesting that when individuals perceive exercise goals as achievable and attribute outcomes to personal effort, their exercise willingness is strongest. In contrast, under conditions of low goal accessibility and external attribution, individuals’ exercise willingness is weakest. This result is consistent with Social Cognitive Theory, which emphasizes the importance of self-efficacy and perceived control over the environment in behavior selection. Specifically, an individual’s self-efficacy (i.e., belief in their ability) and the way they attribute exercise outcomes jointly influence their exercise willingness. When individuals perceive high goal accessibility and believe their effort can determine exercise outcomes, their exercise willingness and participation are more positive (Reilly et al., 2018).

Experiment 2 further examined the effects of attribution style transformation on health beliefs and exercise willingness, particularly the transformation from external attribution to internal attribution. The results showed that transforming from external attribution to internal attribution significantly increased exercise willingness, while transforming from internal attribution to external attribution led to a significant decrease in exercise willingness. Hypotheses H4a and H4b were supported, indicating that attribution style transformation has a significant impact on both exercise willingness and health beliefs. In this way, individuals can enhance their sense of control over exercise outcomes, thereby increasing their motivation to continue engaging in exercise. This finding provides important insights for exercise promotion strategies, suggesting that through appropriate attribution guidance, individuals’ exercise motivation and participation can be significantly influenced.

In summary, this research provides new empirical support for the fields of exercise psychology and the Health Belief Model, particularly in the motivational aspect of exercise willingness. The findings not only validate the independent effects of goal accessibility and attribution style on health beliefs and exercise willingness, but also reveal their interaction effect on exercise willingness. These findings offer theoretical foundations for the design of exercise intervention strategies, particularly with important practical implications for designing effective exercise promotion and health intervention strategies. Specifically, exercise interventions can enhance individuals’ perceptions of goal accessibility and emphasize the decisive role of personal effort in exercise outcomes to increase their exercise willingness and participation.

Although this research achieved a balanced gender distribution during the experimental design phase and avoided overt gender bias in both stimulus presentation and intervention procedures, gender was not systematically incorporated into the core theoretical framework or statistical analysis model. This omission to some extent neglects the potential role of gender as a deep-level moderating factor—particularly within critical pathways such as the acceptance of attributional framing and the formation of health belief systems. Gender identity may influence not only individuals’ sensitivity to intervention content but also systematically shape their cognitive construction of meaning around health-related behaviors.

The primary limitation resulting from this omission lies in the potential presence of a “cross-gender masking effect” in interpreting the intervention outcomes—that is, behind the overall mean values, different gender groups may exhibit intervention responses that are opposite in direction or significantly different in magnitude. However, due to the absence of gender-stratified or model-based analysis, these heterogeneous patterns are obscured by mean-level effects, thereby reducing the research’s ability to identify the critical question of who is more sensitive to the intervention.

Moreover, as a cognitive structure closely related to self-regulation, social norms, and motivational attribution, attribution style may exhibit culturally embedded gender differences in its activation and transformation pathways. For example, internal attribution may be more readily constructed as responsibility-driven among male participants, whereas for female participants, it may be more closely associated with self-worth and socially defined role expectations. If such background biases are not properly addressed, they may introduce interpretive ambiguities in attribution-based interventions, thereby compromising the precision and generalizability of intervention strategies.

Therefore, in the absence of gender-integrated modeling, the conclusions of this research should be extrapolated with caution. Future research should urgently incorporate gender into the theoretical model as an interactive construct, exploring the multi-level moderating mechanism of “goal accessibility × attribution style × gender”. Advanced analytic approaches such as structural equation modeling and latent class analysis (LCA) may also be employed to trace gender-specific cognitive trajectories and behavioral response patterns. Only through the theoretical embedding and methodological integration of gender can researchers more accurately identify the true effectiveness of attribution-based interventions across gender contexts, thereby providing more adaptive and inclusive empirical evidence for the development of health behavior promotion strategies.

## 6. Conclusions

This research systematically investigated the psychological mechanisms through which goal attainability and attribution style—including their interaction and transformation—affect health beliefs and exercise intentions among university students, across two controlled experiments. Experiment 1 focused on the main and interaction effects of goal attainability and attribution style (Hypotheses H1–H4), while Experiment 2 further explored the impact of attributional transformation under varying levels of goal attainability (Hypotheses H5–H8).

Findings from Experiment 1 fully supported H1 and H2: participants with higher perceived goal attainability and those with an internal attribution style exhibited significantly stronger health beliefs and exercise intentions, underscoring the foundational role of attainability perception and self-directed causality in health behavior motivation. H3 was partially supported, as a significant interaction effect was found on exercise intentions but not on health beliefs. Consequently, H4a was not supported, whereas H4b was confirmed: individuals in the high attainability × internal attribution condition reported the highest levels of exercise motivation, while those in the low attainability × external attribution condition reported the lowest.

Results from Experiment 2 confirmed H5a and H5b: individuals who shifted from external to internal attribution exhibited significant improvements in both health beliefs and exercise intentions, whereas those who shifted in the opposite direction showed marked declines. Regarding the moderating role of goal attainability, H6 was partially supported: the interaction between attributional shift and goal attainability significantly affected exercise intentions but not health beliefs. Consistent with this, H7a was not supported, while H7b was validated: participants in the low attainability × internal-to-external shift condition demonstrated the greatest decrease in exercise intentions, while those in the high attainability × external-to-internal shift condition showed the greatest improvement.

Taken together, these findings highlight the synergistic role of cognitive framing (goal attainability) and causal reasoning (attribution style and its transformation) in shaping individuals’ motivation to engage in health-promoting behaviors. By integrating the Health Belief Model and Attribution Theory into a unified experimental framework, this research provides both theoretical insights and practical guidance for developing adaptive, stage-based interventions that promote sustained physical activity.

## 7. New Lines of Research and Limitations of the Research

### 7.1. New Lines of Research

Building on the findings of this study, future research could expand the current framework by incorporating additional psychological and contextual factors that may influence health beliefs and exercise willingness. For instance, the roles of social support, emotional regulation, and cultural norms warrant further investigation, as these variables may interact with goal attainability and attribution style to shape individual responses. Moreover, given that different demographic groups (e.g., by age, gender, or cultural background) may interpret and internalize exercise messages differently, future studies should consider tailoring interventions accordingly. Longitudinal designs could also be employed to examine whether short-term changes in beliefs and intentions translate into sustained behavioral engagement over time.

### 7.2. Limitations of the Researh

Although this research provides important insights into the impact of goal accessibility and attribution style on health beliefs and exercise willingness, there are several limitations. Firstly, the sample primarily consists of Chinese college students, which may limit the generalizability of the findings. Future research could expand to include groups from different age ranges and cultural backgrounds. Secondly, the experimental design had a short duration and did not track participants’ behavioral changes over an extended period. Future studies could adopt longitudinal designs to verify long-term effects. Additionally, the manipulation of attribution style transformation was relatively simplified and may not fully reflect the complexity of real-life situations; future research should consider more refined manipulation techniques. The measurement of goal accessibility relied on advertising images, but different individuals may interpret and perceive these images differently. Future research should employ more precise measurement tools.

Moreover, although the current research employed parametric statistical methods (e.g., ANOVA, *t*-tests) to analyze data collected via seven-point Likert scales, we acknowledge that such scales are technically ordinal in nature. While this approach is commonly accepted in psychological and behavioral research due to its robustness and interpretability, it may introduce limitations in terms of strict statistical assumptions. Therefore, future studies should consider applying non-parametric methods or ordinal regression techniques to more appropriately capture the underlying data structure.

In summary, while this research provides valuable empirical data, further validation is needed with broader samples, long-term tracking, and multidimensional interventions.

## Figures and Tables

**Table 1:** Experiment 1 group distribution (n = 146).

Group	Total Participants	Male Participants	Female Participants
A (High Goal Attainability + Internal Attribution)	30	14	16
B (High Goal Attainability + External Attribution)	37	18	19
C (Low Goal Attainability + Internal Attribution)	40	20	20
D (Low Goal Attainability + External Attribution)	39	20	19
Total	146	72	74

**Table 2:** Descriptive statistics of the pre-test in Experiment 1.

Variable	A (High + Internal)		B (High + External)		C (Low + Internal)		D (Low + External)	
	M	SD	M	SD	M	SD	M	SD
Health Beliefs	3.70	1.43	3.69	1.35	4.11	1.53	4.14	1.51
Exercise Willingness	3.67	1.43	3.91	1.36	4.48	1.43	4.10	1.47
Goal Attainability	3.38	1.62	3.59	1.50	4.16	1.37	4.05	1.59
Attribution Style	3.78	1.41	4.08	1.40	3.78	1.40	4.17	1.37

**Table 3:** Paired samples *t*-test results for post-manipulation goal attainability and attribution style.

Variable	Group	n	M (Difference)	SD (Difference)	t	*p*	95% CI
Goal Attainability	A (High + Internal)	30	−1.10	1.87	−3.22	0.003	(−1.80, −0.40)
	B (High + External)	37	−0.73	1.78	−2.50	0.017	(−1.32, −0.14)
	C (Low + Internal)	40	0.95	1.62	3.70	0.001	(0.43, 1.47)
	D (Low + External)	39	0.85	1.89	2.80	0.008	(0.23, 1.46)
Attribution Style	A (High + Internal)	30	−0.88	2.06	−2.35	0.026	(−1.65, −0.11)
	B (High + External)	37	0.96	1.79	3.26	0.002	(0.36, 1.56)
	C (Low + Internal)	40	−0.73	2.28	−2.02	0.051 ^†^	(−1.45, 0.00)
	D (Low + External)	39	0.82	1.84	2.78	0.008	(0.22, 1.42)

†: marginal significance.

**Table 4:** Independent samples *t*-test results for the effect of goal attainability on health beliefs and exercise intentions.

Variable	Goal Attainability	n	M	SD	t	df	*p*	MD	95%CI
Health Beliefs	High Goal Attainability	67	4.80	1.55	4.11	144	<0.001	0.99	(0.51, 1.46)
	Low Goal Attainability	79	3.81	1.36					
Exercise Willingness	High Goal Attainability	67	4.85	1.12	1.98	142.359	0.049	0.43	(0.001, 0.85)
	Low Goal Attainability	79	4.42	1.48					

**Table 5:** Independent samples *t*-test results for the effect of attribution style on health beliefs and exercise intentions.

Variable	Attribution Style	n	M	SD	t	df	*p*	MD	95%CI
Health Beliefs	Internal Attribution	70	4.53	1.56	2.04	144	0.044	0.51	(0.01, 1.00)
	External Attribution	76	4.02	1.46					
Exercise Willingness	Internal Attribution	70	5.04	1.15	3.79	142.224	<0.001	0.80	(0.38, 1.22)
	External Attribution	76	4.24	1.39					

**Table 6:** Interaction effects of goal attainability × attribution style (two-way ANOVA).

Dependent Variable	Independent Variable	F	*p*	Partial η^2^
Health Beliefs	Interaction (Goal Attainability × Attribution Style)	1.138	0.307	0.503
Exercise Willingness	Interaction (Goal Attainability × Attribution Style)	1.531	0.049	0.577

**Table 7:** The effect of goal attainability and attribution style on exercise intentions (pre-test vs. post-test).

Experimental Group	Pre-Test Exercise Intentions	Post-Test Exercise Intentions	Change Value
A (High Goal Attainability + Internal Attribution)	3.67 ± 1.43	4.87 ± 1.07	1.20
B (High Goal Attainability + External Attribution)	3.91 ± 1.36	4.84 ± 1.17	0.93
C (Low Goal Attainability + Internal Attribution)	4.48 ± 1.43	5.16 ± 1.20	0.68
D (Low Goal Attainability + External Attribution)	4.10 ± 1.47	3.67 ± 1.36	−0.43

Note: Under the interaction effect, Group A showed the largest increase in exercise intentions, followed by Group B. Group C showed a smaller increase, while Group D exhibited a decrease.

**Table 8:** Experiment 2 group distribution (n = 130).

Group	Total Participants	Male Participants	Female Participants
A (High Goal Attainability + Internal Attribution → External Attribution)	31	15	16
B (High Goal Attainability + External Attribution → Internal Attribution)	33	16	17
C (Low Goal Attainability + Internal Attribution → External Attribution)	32	16	16
D (Low Goal Attainability + External Attribution → Internal Attribution)	34	17	17
Total	130	64	66

**Table 9:** Descriptive statistics of the pre-test in Experiment 2.

Variable	A (High + Internal → External)		B (High + External → Internal)		C (Low + Internal → External)		D (Low + External → Internal)	
	M	SD	M	SD	M	SD	M	SD
Health Beliefs	4.484	1.294	3.712	1.447	4.281	1.350	3.662	1.266
Exercise Willingness	4.565	1.257	4.000	1.369	4.438	1.268	3.794	1.533
Goal Attainability	4.274	1.182	3.833	1.555	4.344	1.347	4.382	1.441
Attribution Style	5.484	0.724	2.273	0.601	5.625	0.783	2.250	0.710

**Table 10:** Between-group balance test for pre-test (one-way Analysis of Variance).

Group	Variable	F	*p*
Internal Attribution → External Attribution Group	Health Beliefs	0.370	0.546
	Exercise Willingness	0.159	0.691
	Goal Attainability	0.047	0.828
	Attribution Style	0.551	0.461
External Attribution → Internal Attribution Group	Health Beliefs	0.023	0.880
	Exercise Willingness	0.335	0.564
	Goal Attainability	2.249	0.139
	Attribution Style	0.020	0.888

**Table 11:** Paired samples *t*-test results for goal attainability and attribution style post-manipulation.

Variable	Group	n	M (Difference)	SD (Difference)	t	*p*	95% CI
Goal Accessibility	A (High + Internal → External)	31	−0.71	1.80	−2.19	0.036	(−1.37, −0.05)
	B (High + External → Internal)	33	−0.73	2.22	−1.88	0.069 ^†^	(−1.51, 0.06)
	C (Low + Internal → External)	32	1.00	2.05	2.76	0.01	(0.26, 1.74)
	D (Low + External → Internal)	34	0.87	1.67	3.04	0.005	(0.29, 1.45)
Attribution Style	A (High + Internal → External)	31	1.94	1.45	7.42	0.001	(1.40, 2.47)
	B (High + External → Internal)	33	−2.09	1.57	−7.66	0.001	(−2.65, −1.53)
	C (Low + Internal → External)	32	1.31	1.94	3.83	0.001	(0.61, 2.01)
	D (Low + External → Internal)	34	−2.06	1.85	−6.48	0.001	(−2.71, −1.41)

†: marginal significance.

**Table 12:** Independent sample t-test results for the impact of attribution style transformation on health beliefs and exercise willingness.

Variable	Group	n	M	SD	t	df	*p*	MD	95% Confidence Interval
Health Beliefs	(Internal → External) Group	63	3.460	1.351	−6.460	128.000	<0.001	−1.547	(−2.021, −1.073)
	(External → Internal) Group	67	5.008	1.378					
Exercise Willingness	(Internal → External) Group	63	3.571	1.382	−6.030	128.000	<0.001	−1.436	(−1.907, −0.965)
	(External → Internal) Group	67	5.008	1.333					

**Table 13:** Interaction effects of goal accessibility × attribution style (two-way ANOVA).

Dependent Variable	Independent Variable (Interaction)	F	*p*	Partial η^2^
Health Beliefs	Goal Accessibility × Attribution Style Transformation	0.816	0.772	0.496
Exercise Willingness	Goal Accessibility × Attribution Style Transformation	1.691	0.031	0.671

**Table 14:** The impact of goal accessibility and attribution style transformation on exercise willingness (pre-test vs. post-test).

Experimental Group	Pre-Test Exercise Willingness	Post-Test Exercise Willingness	Change Value
A (High Goal Accessibility + Internal Attribution → External Attribution)	4.565 ± 1.257	4.113 ± 1.377	−0.452
B (High Goal Accessibility + External Attribution → Internal Attribution)	4.000 ± 1.369	5.424 ± 1.024	1.424
C (Low Goal Accessibility + Internal Attribution → External Attribution)	4.438 ± 1.268	3.047 ± 1.187	−1.391
D (Low Goal Accessibility + External Attribution → Internal Attribution)	3.794 ± 1.533	4.603 ± 1.481	0.809

Note: Under the interaction effect, Group B showed the greatest increase in exercise willingness, followed by Group D. Group C showed a slight decrease, while Group D showed a significant decrease.

## Data Availability

The data supporting the findings of this study are openly available in the Open Science Framework (OSF) at https://osf.io/9skwm/?view_only=68615a4b2e134f2eb9d5271b9b55187d (accessed on 26 May 2025).

## Appendix A

**Table A1:** Measurement tools used in the experiment (applicable to both Experiment 1 and Experiment 2).

Scale Name	Item	Response						
Demographic Information	1. What is your gender?	Male/Female						
	2. How old are you (in years)?	Fill in						
Goal Accessibility Scale	1. I believe the effects of exercise can be quickly observed	1	2	3	4	5	6	7
	2. I think exercise requires long-term commitment to see significant results.	1	2	3	4	5	6	7
Attribution Style Scale	1. My exercise outcomes primarily depend on my effort.	1	2	3	4	5	6	7
	2. External factors, such as environment, weather, and genetics, are more important than personal effort.	1	2	3	4	5	6	7
Health Belief Scale	1. I believe that exercise can significantly improve my health condition	1	2	3	4	5	6	7
	2. Even without short-term effects, exercise is still important for my health.	1	2	3	4	5	6	7
Exercise Willingness Scale	1. I plan to exercise at least three times per week in the future, if given the opportunity	1	2	3	4	5	6	7
	2. I am willing to engage in more physical exercise.	1	2	3	4	5	6	7
